# Visual Exploration Area in Neglect: A New Analysis Method for Video-Oculography Data Based on Foveal Vision

**DOI:** 10.3389/fnins.2019.01412

**Published:** 2020-01-22

**Authors:** Brigitte Charlotte Kaufmann, Samuel Elia Johannes Knobel, Tobias Nef, René Martin Müri, Dario Cazzoli, Thomas Nyffeler

**Affiliations:** ^1^Perception and Eye Movement Laboratory, Departments of Neurology and BioMedical Research, Inselspital, Bern University Hospital, University of Bern, Bern, Switzerland; ^2^Neurocenter, Luzerner Kantonsspital, Lucerne, Switzerland; ^3^Gerontechnology and Rehabilitation Group, ARTORG Center for Biomedical Engineering Research, University of Bern, Bern, Switzerland

**Keywords:** visual neglect, video-oculography, visual exploration area, visual attention, foveal vision, visual exploration behavior, mean gaze position, free visual exploration

## Abstract

Video-oculography during free visual exploration (FVE) is a valuable tool to evaluate visual attention spatial allocation in neglect patients after right-hemispheric stroke. In conventional FVE analyses, the position of a visual fixation is conceived as a single point in space. Here, we describe a new complementary method to analyze FVE data based on foveal vision, leading to an accurate estimate of the portion of the picture that was effectively explored. In 15 neglect patients and 20 healthy controls, visual exploration areas (i.e., considering 1° visual angle around every single fixation) were computed. Furthermore, the proportion of single and overlapping fixations was analyzed. Overlapping fixations were further categorized into capture fixations (successive overlapping fixation, putatively reflecting problem of disengagement) and re-capture fixations (temporally distant overlapping fixations, putatively reflecting spatial working memory deficits). The results of this new analysis approach were compared to the ones of conventional approaches. Conventional analyses showed the typical visual attention deficits in neglect patients versus healthy controls: significantly less fixations and time spent within the left and significantly more fixations and time spent within the right screen half. According to the results of our new approach, patients showed a significantly smaller visual exploration area within the left screen half. However, the right visual exploration area did not differ between groups. Furthermore, in neglect patients, the proportion of overlapping fixations within the right screen half was significantly higher than within the left screen half, as well as significantly higher than in healthy controls within either screen halves. Whereas neglect patients showed significantly more capture fixations than healthy controls, the number of re-capture fixations did not differ between groups. These results suggest that, in neglect patients, the efficiency of visual exploration is also reduced within the right screen half and that impaired disengagement might be an important mechanism leading to overlapping fixations. Our new analysis of the visual exploration area, based on foveal vision, may be a promising additional approach in visual attention research. It allows to accurately measure the portion of the picture that was effectively explored, disentangle single from overlapping fixations, and distinguish between capture and re-capture fixations.

## Introduction

Unilateral spatial neglect is characterized by the failure to attend or respond to the contralesional hemispace (Heilman et al., 1993). After stroke, neglect has been reported to occur in 43–80% of patients with a right-hemispheric lesion and in 20–62% of patients with a left-hemispheric lesion (Stone et al., 1991; Azouvi et al., 2002; Ringman et al., 2004). Recently, video-oculography during free visual exploration (FVE) has been shown to be a valuable and reliable tool to evaluate visual attention allocation in neglect patients (Pflugshaupt et al., 2004; Ptak et al., 2009; Cazzoli et al., 2010, 2011; Osandón et al., 2012; Fellrath and Ptak, 2015; Delazer et al., 2018; Ohmatsu et al., 2019; Paladini et al., 2019).

Several exploration parameters have been established as typical neglect indicators during FVE. For instance, the spatial distribution of visual fixations along the horizontal axis (assessed by means of the mean gaze position and the mean number of fixations within a given portion of the exploration field) is typically shifted toward the ipsilesional screen half in neglect patients as compared to healthy controls. Also, an ipsilesional bias is observed when the time spent within each screen half or the position of the leftmost fixation is considered (e.g., Dijkerman et al., 2003; Pflugshaupt et al., 2004; Cazzoli et al., 2009; Ohmatsu et al., 2019; Paladini et al., 2019).

In the present paper, we describe a new analysis approach to assess the spatial distribution of visual fixations during FVE. The spatial position of a visual fixation is generally conceived as a single point in space (i.e., considering the single coordinates of the center of the fixation in the calculations). However, processing of visual information during a visual fixation entails not only its central point but also a visual angle of 1° around it (i.e., foveal vision; Miellet et al., 2009; Strasburger et al., 2011). By computing these circular areas around every single fixation point, one could assess their spatial summation and/or overlap, thus leading to an accurate estimate of the portion of the picture that was effectively explored. Such a procedure could be able to detect even subtle changes at the border of the fixation distribution. Indeed, clinical observations (e.g., comparing individual fixation plots before and after a given therapy) often show that the field of visual exploration in neglect patients may have slightly expanded toward the left. In those patients, however, the results of analyses based on a dichotomous categorization (i.e., in left and right screen halves) often remain unchanged. A further advantage of the proposed approach, based on fixation areas rather than points, could be that the percentage of single and overlapping fixations can easily be assessed. Indeed, it has recently been shown that neglect patients produce several fixations on nearby coordinates during FVE, whereas this phenomenon is significantly less pronounced in healthy subjects (Paladini et al., 2019). Hence, particularly, in neglect patients, the fixation areas may overlap. In turn, this can have an influence on the portion of the picture that is effectively explored (e.g., a certain number of overlapping fixations can lead to a smaller exploration area in comparison to the same number of non-overlapping single fixations).

Here, we apply this methodological approach to assess the area of effective visual exploration during FVE in neglect patients and age-matched healthy controls. We then compare the results of this approach with the ones of the most commonly used indicators to measure the spatial distribution of visual fixations in FVE (mean gaze position, mean number of fixations, mean cumulative fixation duration, and leftmost fixation).

## Materials and Methods

### Subjects

Fifteen patients (aged between 56 and 89 years, mean = 72.20, *SD* = 9.563; five female) with left-sided visual neglect after a first, ischemic or hemorrhagic, right-hemispheric stroke (subacute phase; days since stroke between 4 and 34, mean = 16.467, *SD* = 8.459) and 20 age-matched healthy controls (aged between 41 and 89 years, mean = 66.60, *SD* = 10.102; nine female) were included in the study (no significant age difference between healthy controls and neglect patients; *t*(33) = −1.660, *p* = 0.106, two-tailed *t*-test). All participants had normal or corrected-to-normal visual acuity.

Patients were included in the study if they showed neglect in at least one of the following neuropsychological paper-pencil tests: Letter Cancelation test [neglect cutoff: center of cancelation (CoC) > 0.083; Weintraub and Mesulam, 1988], Bells Test (neglect cutoff: CoC > 0.081; Rorden and Karnath, 2010), Random Shape Cancelation Test (neglect cutoff: CoC > 0.081; Weintraub and Mesulam, 1988), Line Bisection Test (neglect cutoff: relative rightward deviation of > 11%; Wilson et al., 1987), or Five-Point Test (neglect cutoff: CoC > 0.081; Regard et al., 1982; Kaufmann et al., 2018). Furthermore, all patients presented with neglect during their everyday behavior, as assessed by means of the Catherine Bergego Scale (CBS) (neglect cutoff: CBS ≥ 1; mean = 11.4, *SD* = 7.51; Azouvi et al., 2003). Table 1 shows the patients’ individual demographic data (age range, time since stroke, and absence/presence of visual field defects, as assessed by means of Goldmann perimetry), as well as their scores in the different neuropsychological paper-pencil tests and in the CBS.

**TABLE 1:** Demographic and clinical data of neglect patients.

**Patient code**	**Age-range**	**Time since stroke (days)**	**CBS (score)**	**Five point (CoC)**	**Letter cancelation (CoC)**	**Line bisection (relative deviation in %)**	**Bells (CoC)**	**Random shape (CoC)**	**Visual field defects**
Pat_ 01	61–65	17	**7**	**0.41**	**0.65**	7.76	**0.64**	**0.876**	No
Pat_ 02	81–85	13	**9**	**0.55**	–0.01	4.88	**0.16**	0.059	No
Pat_ 03	76–80	20	**4**	0.08	0.05	7.01	**0.13**	–0.016	No
Pat_ 04	81–85	12	**6**	**0.98**	**0.20**	**27.20**	**0.16**	0.002	No
Pat_ 05	56–60	32	**16**	**0.93**	**0.46**	**94.82**	**0.15**	0.021	No
Pat_ 06	61–65	18	**14**	**0.12**	**0.69**	**53.18**	**0.35**	**0.709**	Not available
Pat_ 07	66–70	7	**25**	**0.57**	**0.79**	**93.83**	**0.97**	**0.919**	Hemianopia
Pat_ 08	71–75	4	**19**	**0.98**	**0.69**	**20.21**	**0.79**	**0.854**	No
Pat_ 09	61–65	16	**3**	**0.32**	**0.58**	4.06	**0.16**	0.000	No
Pat_ 10	61–65	17	**11**	–0.07	–0.06	**16.58**	**0.15**	0.049	Inferior quadrantanopia (central 20° intact)
Pat_ 11	76–80	34	**26**	**0.92**	**0.23**	**18.93**	**0.72**	**0.380**	Inferior quadrantanopia (central 20° intact)
Pat_ 12	66–70	8	**12**	–0.03	**0.28**	**28.87**	–0.02	0.032	No
Pat_ 13	76–80	12	**4**	**0.31**	0.03	–2.69	0.04	0.034	No
Pat_ 14	86–90	24	**12**	**0.19**	0.01	1.33	0.05	0.004	No
Pat_ 15	71–75	13	**3**	**0.49**	0.03	**15.10**	**0.12**	0.040	No

*Table shows patients’ individual demographic data (age range, time since stroke in days, and presence/absence of visual field defects, as assessed by means of Goldmann perimetry), their individual neglect severity score in the activities of daily living as assessed with the Catherine Bergego Scale (CBS; range 0–30, neglect cutoff CBS ≥ 1), as well as their individual scores for the following neuropsychological tests: Five Point Test [neglect cutoff: Center of Cancelation (CoC) > 0.081; Kaufmann et al., 2018], Letter Cancelation Test (neglect cutoff: CoC > 0.083; Rorden and Karnath, 2010), Line Bisection (neglect cutoff: relative rightward deviation of >11%; Wilson et al., 1987), Bells Test (neglect cutoff: CoC > 0.081; Rorden and Karnath, 2010), and Random Shape Cancelation test (neglect cutoff: CoC > 0.081; Weintraub and Mesulam, 1988). Individual test scores indicating neglect are displayed in bold type.*

Ethical approval was provided by the Ethics Committee Nordwest and Zentralschweiz (EKNZ), Switzerland. The study was carried out in accordance with the latest version of the Declaration of Helsinki.

### Video-Oculography

In all participants, video-oculography was used to assess visual fixation behavior during FVE, a paradigm that has reliably been used to assess visual attention allocation in space (Pflugshaupt et al., 2004; Ptak et al., 2009; Cazzoli et al., 2010, 2011; Osandón et al., 2012; Fellrath and Ptak, 2015; Delazer et al., 2018; Paladini et al., 2019). Hereby, 12 pictures of natural scenes or urban public places (full color, resolution of 1,200 × 900 pixels) and their 12 mirrored versions (mirrored along the vertical axis) were presented on a computer screen (Paladini et al., 2019). Each of the pictures was presented for 7 s. To enforce a common starting point of visual exploration for all participants, pictures were preceded by a central black fixation cross on a gray background, displayed for 1 s. All participants were instructed to freely explore the pictures and to fixate on the central fixation cross presented between pictures. During video-oculography, participants were seated in front of the computer screen. Their head was positioned on a chin-and-forehead rest to ensure that their mid-sagittal plane was aligned with the middle of the screen at a constant distance of 68 cm (resulting in a viewing angle of 27° × 21°) and to minimize head movements.

Eye movements were recorded using a remote, infrared-based, video eye-tracking system (EyeLink 1000 Plus System, SR Research, Ottawa, ON, Canada; sampling rate of 1,000 Hz; gaze position accuracy of typically 0.25°–0.50°, depending on calibration accuracy; spatial resolution of typically 0.01°). The system was calibrated to the participants’ individual eye movements prior to the experiment by means of a 3 × 3-point grid. Only fixations with a duration between 100 and 2,000 ms were retained for offline data analysis (Salthouse and Ellis, 1980; Carpenter, 1988), which resulted in the exclusion of 7.89% of all fixations.

### Conventional Analyses of Free Visual Exploration Data

Four conventional analyses, aimed at quantifying neglect severity, were performed: (1) mean horizontal gaze position, reflecting the center of mass of the spatial distribution of visual fixations on the horizontal axis during FVE (e.g., Paladini et al., 2019). The mean horizontal gaze position was calculated in degrees of visual angle from the center of the picture (0 = perfectly aligned with the horizontal middle of the picture; negative values = mean horizontal gaze position within the left half of the screen; positive values = mean horizontal gaze position within the right half of the screen); (2) horizontal position of the leftmost visual fixation (in degrees of visual angle from the center of the picture), reflecting the maximum leftward extensions of the FVE field (e.g., Dijkerman et al., 2003); (3) mean number of fixations in the left and right halves of the screen, reflecting the numerical distribution of fixations in the left and right FVE fields (e.g., Pflugshaupt et al., 2004; Paladini et al., 2019); and (4) mean cumulative fixation duration in the left and right halves of the screen, reflecting the time spent in the left and right FVE fields (e.g., Pflugshaupt et al., 2004; Cazzoli et al., 2009).

For the conventional analyses, all variables were first computed for every picture and participant, then averaged over all pictures per participant. Each corresponding variable was compared between healthy controls and neglect patients using an independent-samples *t*-test (two-tailed) or, when appropriate, by means of a 2 × 2 mixed model ANOVA with the factors group (two levels: healthy controls, neglect patients) and screen half (two levels: left and right halves of the screen). Bonferroni-corrected *post hoc t*-tests were used to compare the results between the different combinations of factor levels.

For all statistical tests, the significance level of α = 5% was used.

### New Analysis Approach of Free Visual Exploration Data

**FIGURE 1:**
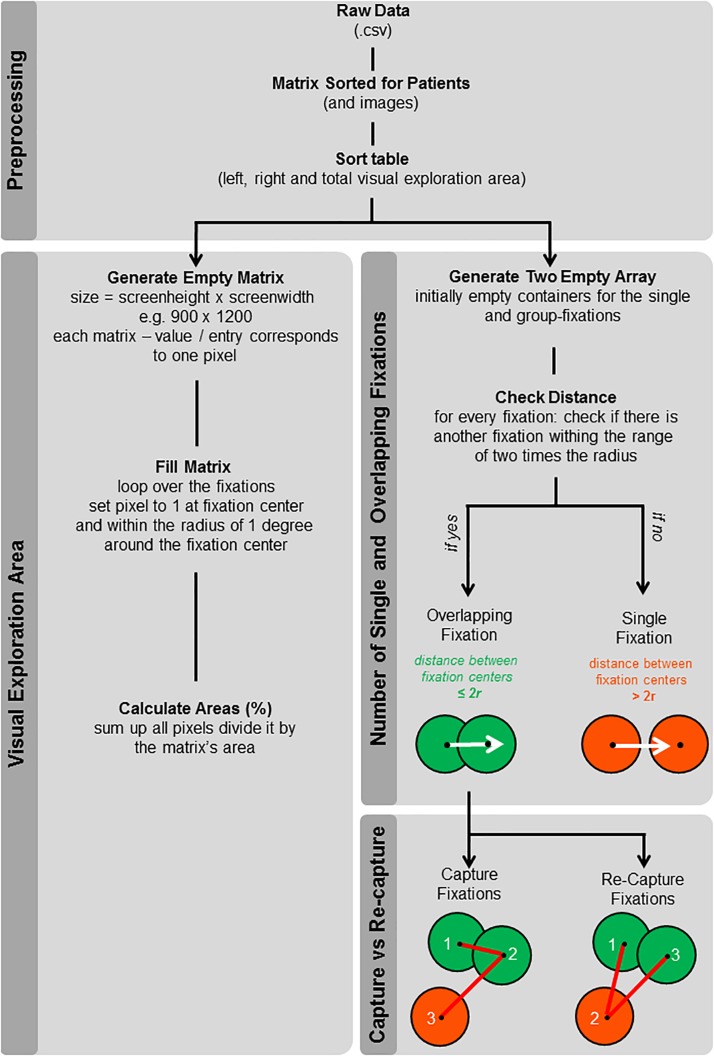
Schematic working path for the calculation of the new analyses: visual exploration area, number of overlapping and single fixations, and number of capture and re-capture fixations. The flowchart shows the structure of the MATLAB code used to calculate the visual exploration area as well as the number of overlapping and single fixations for the picture-wise and the cumulative analysis. Single fixations (dark orange) are fixations for which no other fixation took place within the range of twice the radius from their fixation center. Overlapping fixations (green) show an intersection with at least one area of another fixation. Additionally, overlapping fixations were categorized into capture fixations (i.e., consecutive fixations at the same spatial location) and re-capture fixations (i.e., when participants disengage from a given spatial location and come back to it in the later course of time).

Generally, fixations during FVE are conceived as a single point, with *x*- and *y*-coordinates, in a given reference frame, for example, a picture presented during the task. However, a fixation entails not only its central point (i.e., the aforementioned *x*- and *y*-coordinates) but processing of visual information takes place in an area around it. This area, corresponding to foveal vision (defined as vision using the part of the central retina with the maximum visual acuity, constantly displaced across fixations during FVE), consists of the central 1.5–2° of the visual field (Miellet et al., 2009; Strasburger et al., 2011). Thus, by virtually drawing a circle with a radius of 1° visual angle around each fixation, and in a second step, by summing these single fixation areas, it is possible to calculate the size of the total effective visual exploration area. Since during FVE, several fixations may take place on nearby coordinates (Paladini et al., 2019), it is also possible to assess the number of overlapping fixations and single (i.e., non-overlapping) fixations. Furthermore, overlapping fixations can be classified into (a) capture fixations (successive overlapping fixation, putatively reflecting problem of impaired disengagement) and (b) re-capture fixations (i.e., temporally distant overlapping fixations, putatively reflecting spatial working memory deficits; in this case, subjects disengage from a spatial location and come back to it in the later course of time); see also illustration in Figure 1 and further explanation in section “Coding of the Visual Exploration Area and Fixation Categorization” and section “Statistical Analyses of Visual Exploration Area and Fixation Categorization Results”).

Detailed information concerning the coding of the visual exploration area and fixation categorization (i.e., overlapping or single fixations) is provided in the section “Coding of the Visual Exploration Area and Fixation Categorization.”

#### Coding of the Visual Exploration Area and Fixation Categorization

Coding of the visual exploration area and fixation categorization were performed by means of a customized MATLAB script (MatWorks Inc., Natick, MA, United States). The raw data are imported and fixations are formatted and sorted according to participants, pictures, and location (right half of the screen, left half of the screen, and total screen). Then, the script provides two independent options to analyze FVE data: (A) the visual exploration area and (B) the number of overlapping and single fixations. Figure 1 shows the schematic working path for the calculation of the new analyses.

In (A), a matrix with the size of 900 × 1,200 (corresponding to the pixels of the presented picture) is created and filled with zeros. The zero value of a given cell of the matrix is then replaced by a one if the cell itself lies within the radius of 1° visual angle (43 pixels) from the coordinates of a given fixation. If a given cell lies within the radius of several fixations, this cell is still tagged only once (i.e., a given cell can either have the value of 0 = not within the radius of any fixation or the value of 1 = within the radius of at least one fixation). In a final step, the visual exploration area is calculated by dividing the sum of all ones in the requested part of the screen (left half of the screen, right half of the screen, or total screen) by the total size of the matrix (Figure 1, left part). In (B), the script allows to additionally differentiate between two categories of fixations, that is, overlapping fixations and single fixations (Figure 1, right part). Single fixations are defined as the ones for which no other fixation took place within the range of twice the radius from the respective fixation center (2^∗^*r* = 2°; Figure 1, lower right part, definition of single fixation highlighted in dark orange). Thus, the area around a single fixation does not show an intersection with any other fixation area. Afterward, the number of overlaying fixations is calculated by subtracting the number of single fixations from the total number of fixations (Figure 1, lower right part, definition of overlapping fixation highlighted in green).

Both parts of the script, A and B, can be performed for cumulative analyses (i.e., over all 12 pictures and their 12 mirrored versions) as described above. For picture-wise analyses, an additional loop is implemented in the script, allowing to compute the exploration area, as well as the number of single and overlapping fixations, for every picture and participant. Furthermore, overlapping fixations can be classified in capture fixations (successive overlapping fixation, i.e., fixations that occur at a spatial location within 1° of their respective, immediately preceding fixation) and re-capture fixations (temporally distant overlapping fixations, in this case, subjects disengage from a spatial location and come back to it in the later course of time, i.e., fixations that occur within 1° of a previous fixation).

The average overall pictures can then be manually computed for every participant.

**FIGURE 2:**
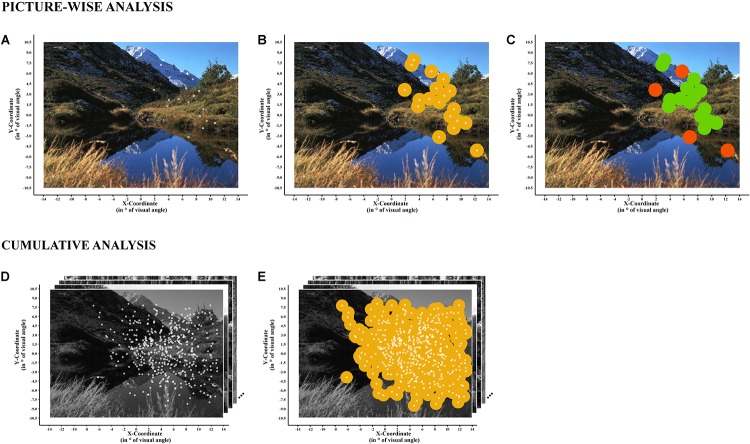
Example of the picture-wise analysis (top) and cumulative analysis (bottom) of the visual exploration area and of the number of overlapping versus single fixations. Picture-Wise Analysis: **(A)** The gaze plot of a single neglect patient for a single exemplary picture, shown in the background. **(B)** Illustrates the overlay of fixations and visual exploration areas. The visual exploration area is calculated based on foveal vision (*r* = 1°, yellow). In **(C)**, the computation of the number of overlapping fixations (*n* = 15, in green) and the number of single fixations (*n* = 4, in dark orange) are illustrated. Cumulative Analysis: **(D)** The cumulative gaze plot, that is, combining all fixations taking place during the whole experiment (over 12 pictures and their 12 mirrored versions) for a single patient. **(E)** The overlay of fixations and visual exploration areas calculated based on foveal vision (*r* = 1°). Exemplary pictures (here in black and white for the purpose of illustration; in full color during the experiment) are shown in the background.

Figure 2 illustrates the output based on the MATLAB script for the picture-wise analyses (top) and the cumulative analyses over all 24 pictures (bottom).

#### Statistical Analyses of Visual Exploration Area and Fixation Categorization Results

We evaluated participants’ mean visual exploration area (picture-wise analysis, in % of the total picture surface) as well as their cumulative visual exploration area (i.e., cumulative analysis over all 24 pictures in %, see above). The visual exploration areas (picture-wise and cumulative) were compared between healthy controls and neglect patients using an independent-samples *t*-test (two-tailed). Furthermore, the visual exploration areas were compared across the left and right halves of the screen between the two groups (healthy controls, neglect patients) by means of a 2 × 2 mixed-model ANOVA with the factors group (two levels: healthy controls, neglect patients) and screen half (two levels: left, right).

As a further analysis, we were interested in assessing whether visual exploration would take place in clusters of overlapping fixations or rather in single fixations. Hereby, the number of overlapping fixations, as well as the number of single fixations, was calculated per picture (for the left and the right halves of the screen) and averaged per participant. The mean number of overlapping and single fixations was analyzed using a 2^∗^2^∗^2 mixed-model ANOVA with the factors group (two levels: healthy controls, neglect patients), screen half (two levels: left, right), and fixation category (two levels: single fixations, overlapping fixations). Additionally, the proportion of overlapping fixations (i.e., number of overlapping fixations divided by number of single fixations) was analyzed by means of a 2 × 2 mixed-model ANOVA with the factors group (two levels: healthy controls, neglect patients) and screen half (two levels: left, right).

Bonferroni-corrected *post hoc t*-tests were used to compare the results between the different combinations of factor levels.

In a subsequent analysis, we aimed to assess which proportion of overlapping fixations reflects a form of successive fixations on one spatial location (capture fixations; putatively reflecting problem of disengagement) or of temporally distant fixations, that is, whether participants disengage from a spatial location and come back to it in the later course of time (re-capture fixations; putatively reflecting spatial working memory deficits). Therefore, the number of capture fixations and of re-capture fixations was compared between healthy controls and neglect patients by means of a 2 × 2 mixed-model ANOVA with the factors group (two levels: healthy controls, neglect patients) and overlapping fixations category (two levels: capture fixation, re-capture fixation). Additionally, a qualitative illustration was made to show the temporal dynamics of capture and re-capture fixations over the first 20 fixations (Figure 7B).

## Results

### Conventional Analyses of Free Visual Exploration Data

#### Mean Gaze Position

**FIGURE 3:**
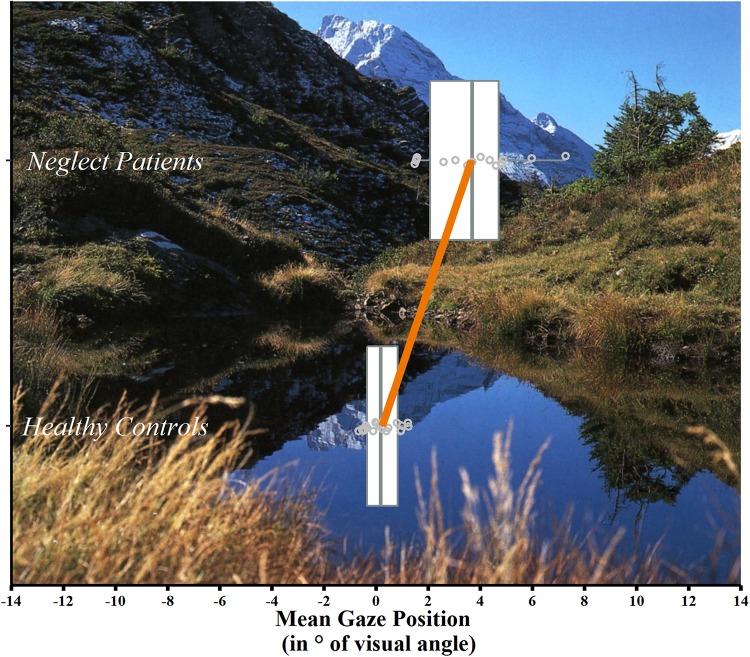
Mean gaze position per group. Box-and-whisker plots of the mean horizontal gaze position (in° of visual angle) in neglect patients (*n* = 15, top) and healthy controls (*n* = 20, bottom). Each box represents the lower (Q1) to the upper (Q3) quartiles, with whiskers extending to the minimum and maximum of 1.5 times the interquartile range; the gray vertical lines represent the median. Additionally, mean values of the two groups are indicated by the endpoints of the orange line and individual values by gray dots. An exemplary picture, used in the FVE paradigm, is shown in the background.

Neglect patients showed a significant rightward horizontal shift in their mean gaze position as compared to healthy controls (neglect patients = + 3.652°, *SD* = 1.741°; healthy controls = + 0.231°, *SD* = 0.621°; *t*(16.686) = −7.275, *p* < 0.000, two-tailed; Figure 3). Cohen’s effect size (*r* = 0.8719) indicated a very strong effect.

#### Leftmost Fixation

Compared to healthy controls, neglect patients showed a significant rightward shift in the mean horizontal coordinates of their leftmost fixation (healthy controls: mean = −9.959°, *SD* = 1.533°; neglect patients: mean = −3.482°, *SD* = 2.759°; *t*(33) = −8.859, *p* < 0.001, two-tailed). Cohen’s effect size (*r* = 0.8390) indicated a very strong effect.

#### Mean Number of Fixations

A mixed-model ANOVA revealed a significant main effect for both factors group (F _33_,_1_ = 14.3214, *p* = 0.001) and screen half (*F*_33_,_1_ = 78.857, *p* < 0.001) as well as a significant interaction between these factors (group × screen half: *F*_33_,_1_ = 64.143, *p* < 0.001). The effect size for the interaction showed a large effect (η^2^ = 0.531).

**FIGURE 4:**
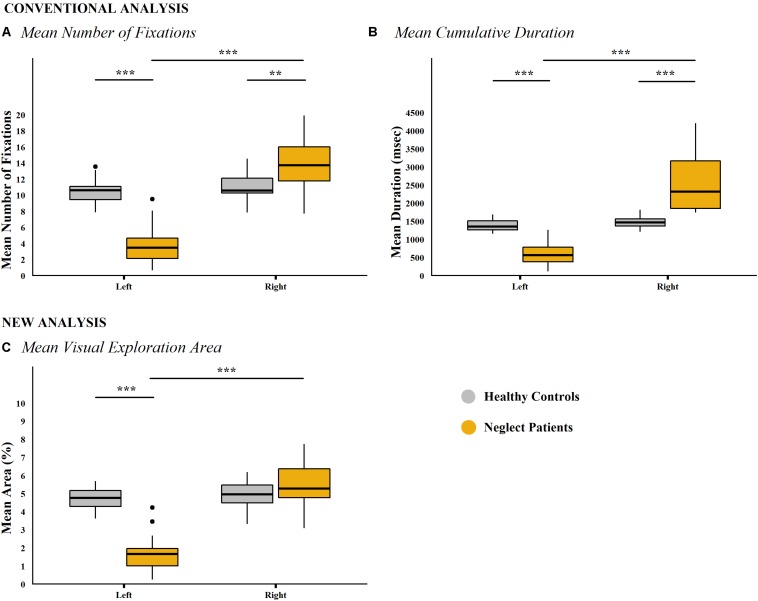
Conventional analyses and new analysis per group and screen half (picture-wise analysis). Box-and-whisker plots for the conventional analysis approaches: **(A)** Mean number of fixations. **(B)** Mean cumulative fixation duration per group and screen half. New analysis approach: **(C)** Mean visual exploration area per group and screen half. Results of neglect patients (*n* = 15) are depicted in yellow, those of healthy controls (*n* = 20) in gray. In contrast to conventional analysis approaches, analysis of the mean visual exploration area in the right half of the screen did not reveal any difference between neglect patients and healthy controls, that is, the effectively explored area in the right half of the screen did not differ between groups. Significant differences are depicted by asterisks (***p < 0.001, **p < 0.01, and *p < 0.05). Median values per group are indicated by the horizontal black line in each box. Each box represents the lower (Q1) to the upper (Q3) quartiles, with whiskers extending to the minimum and maximum of 1.5 times the interquartile range. Outliers are indicated by black dots.

*Post hoc* tests showed, as expected, the characteristic spatial difference between left and right halves of the screen in neglect patients (number of fixations left = 3.848, *SD* = 2.603; number of fixations right = 13.859, *SD* = 3.242; *p* < 0.001). In healthy controls, no significant difference between the screen halves was found (left = 10.495, *SD* = 1.522; right = 11.012, *SD* = 1.706; *p* = 0.909; Figure 4A).

Further typical differences between neglect patients and healthy controls were observed. Within the left half of the screen, neglect patients showed significantly less fixations compared to healthy controls (*p* < 0.001), whereas the inverse pattern was found within the right half of the screen (*p* = 0.002).

#### Mean Cumulative Fixation Duration

A mixed-model ANOVA showed a significant main effect for both factors group (*F*_33_,_1_ = 4.933, *p* = 0.033) and screen half (*F*_33_,_1_ = 68.512, *p* < 0.001), as well as a significant interaction between these factors (group × screen half: *F*_33_,_1_ = 54.072, *p* < 0.001). The effect size for the interaction showed a large effect (η^2^ = 0.563).

The aforementioned characteristic pattern of neglect was also clearly evident in *post hoc* analysis of the time spent in either screen half. Neglect patients spent less time exploring the left compared to the right half of the screen (time left = 603.06 ms, *SD* = 323.066 ms; time right = 2,552.14 ms, *SD* = 790.321 ms; *p* < 0.001), whereas this spatial bias was not found in healthy controls (time left = 1,376.29 ms, *SD* = 160.686 ms; time right = 1,491.36 ms, *SD* = 157.44 ms; *p* = 0.894, Figure 4B).

Moreover, compared to healthy controls, neglect patients spent significantly less time exploring the left half of the screen (*p* < 0.001) but more time exploring the right half of the screen (*p* < 0.001).

### New Analysis Approach of Free Visual Exploration Data

#### Mean Visual Exploration Area (Picture-Wise Analysis)

A mixed-model ANOVA showed a significant main effect for both factors group (*F*_33_,_1_ = 29.407, *p* < 0.001) and screen half (*F*_33_,_1_ = 84.983, *p* < 0.001). More precisely, neglect patients showed a significantly smaller mean visual exploration area compared to healthy controls (neglect patients = 7.159%, *SD* = 1.479%; healthy controls 9.633%, *SD* = 1.219%). Furthermore, the interaction between these factors (group × screen half; *F*_33_,_1_ = 68.967, *p* < 0.001) revealed a significant and large effect (η^2^ = 0.496).

*Post hoc* tests showed that the mean visual exploration area was significantly smaller in the left compared to the right screen half in neglect patients (left = 1.693%, *SD* = 1.091%; right = 5.466%, *SD* = 1.171%; *p* < 0.001) but not in healthy controls (left = 4.718%, *SD* = 0.618%; right = 4.915%, *SD* = 0.822%; *p* = 0.999; Figure 4C). Furthermore, neglect patients showed a significantly smaller visual exploration area in the left half of the screen compared to healthy controls (*p* < 0.001). However, in contrast to the above described conventional analyses, the visual exploration area in the right half of the screen did not differ between neglect patients and healthy controls (*p* = 0.582).

#### Overall Visual Exploration Area (Cumulative Analysis)

**FIGURE 5:**
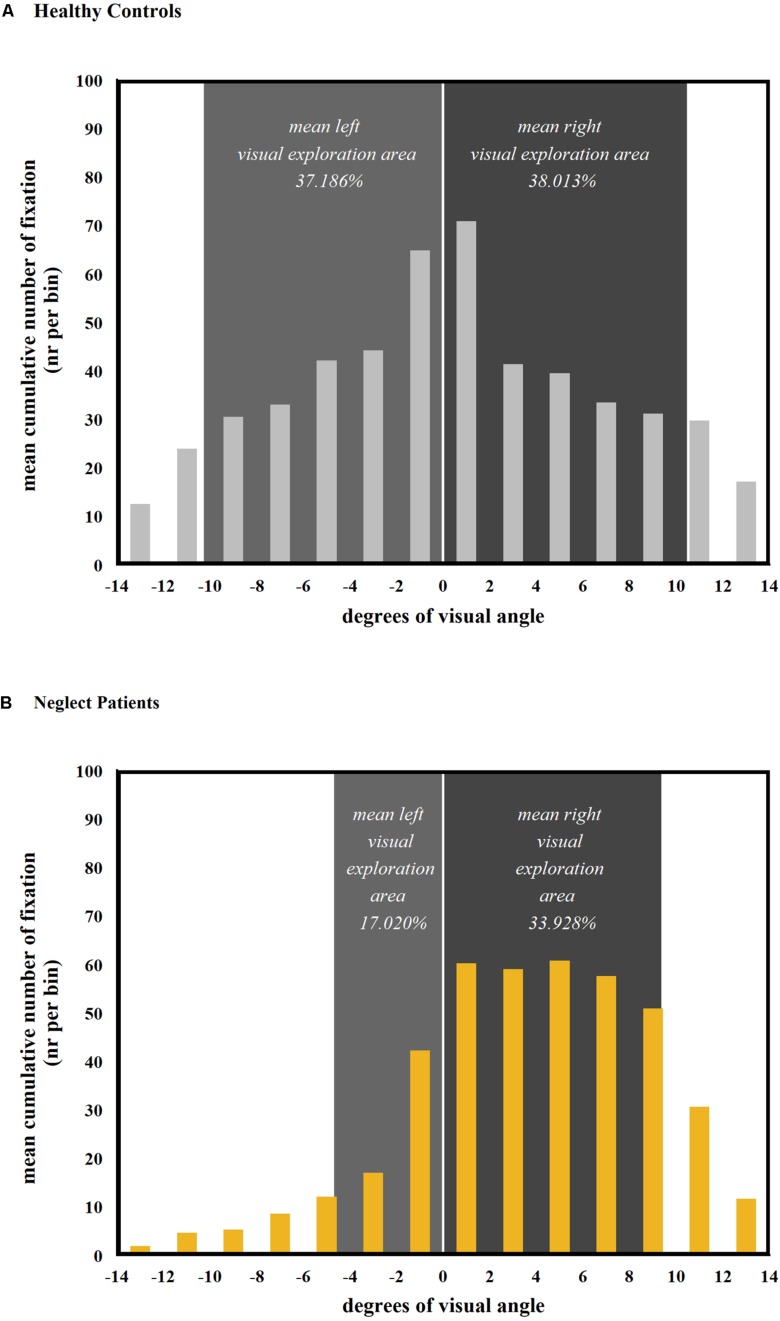
Comparison of mean visual exploration area and spatial distribution of the number of fixations per group and screen half (cumulative analysis over all 24 pictures). The histograms show the spatial distribution of fixations over horizontal bins of 2° visual angle. The mean cumulative visual exploration area for the left and the right halves of the screen is depicted in light gray and dark gray, respectively. Even though neglect patients **(B)** produced more fixations within the right half of the screen, their mean right visual exploration area was slightly smaller than the one of healthy controls [**A**; see statistical comparisons in section “Mean Number of Fixations” and section “Overall Visual Exploration Area (Cumulative Analysis)”].

Similar results were also found for the cumulative analysis of the overall visual exploration area. A mixed-model ANOVA revealed a significant main effect for both factors GROUp (*F*_33_,_1_ = 42.475, *p* < 0.001) and screen half (*F*_33_,_1_ = 75.374, *p* < 0.001). Neglect patients showed a significantly smaller overall visual exploration area than healthy controls (neglect patients = 50.948%, *SD* = 12.847%); healthy controls = 75.199%, *SD* = 9.193%; Figure 5). Furthermore, a significant interaction between these factors was found (group × screen half; *F*_33_,_1_ = 61.975, *p* < 0.001). The effect size for the interaction showed a large effect (η^2^ = 0.303).

As confirmed by *post hoc* tests, the overall visual exploration area was significantly smaller in the left compared to the right half of the screen in neglect patients (left = 17.020%, *SD* = 9.327%; right = 33.928%, *SD* = 5.644%; *p* < 0.001), but no significant difference was found in healthy controls (left = 37.186%, *SD* = 4.401%; right = 38.013%, *SD* = 5.213%; *p* = 1.00; Figure 5).

Within the left half of the screen, neglect patients showed a significantly smaller overall visual exploration area than healthy controls (*p* < 0.001). In line with the picture-wise analysis, no group difference was found within the right half of the screen (*p* = 0.781).

A closer inspection revealed that even though the right visual exploration area did not differ between patients and healthy controls, neglect patients produced significantly more fixations in the right half of the screen than healthy controls (see section “Mean Number of Fixations”). This dissociation between the spatial distribution of fixations and the visual exploration area (Figure 5) may be explained by an increase of overlapping fixations in neglect patients during exploration of the right half of the screen. Therefore, in a next analysis, the number of overlapping and single fixations was compared between the two halves of the screen, as well as between neglect patients and healthy controls.

#### Overlapping and Single Fixations

##### Absolute number of overlapping and single fixations

A mixed-model ANOVA showed significant main effects for all factors, that is, group (*F*_33_,_1_ = 14.34, *p* = 0.001), screen half (*F*_33_,_1_ = 78.92, *p* < 0.001), and fixation category (*F*_33_,_1_ = 52.12, *p* < 0.001). The interaction group × screen half × fixation category was also significant (*F*_33_,_1_ = 34.35, *p* < 0.001), with a large effect size (η^2^ = 0.159).

**FIGURE 6:**
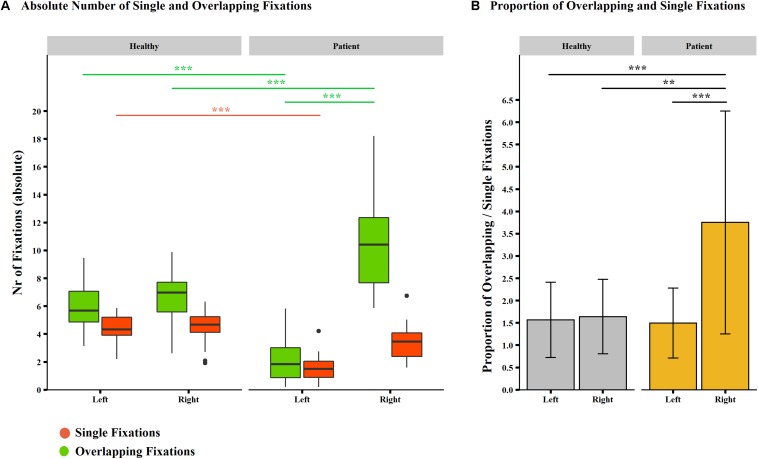
Absolute number and proportion of overlapping and single fixations per group and screen half (picture-wise analysis. **(A)** Box-and-whisker plots of the mean number of fixations per fixation category (overlapping fixations, in green; single fixations, in dark orange) and screen half in neglect patients (*n* = 15, right-hand side) and healthy controls (*n* = 20, left-hand side). Median values per group are indicated by the horizontal black line in each box. Each box represents the lower (Q1) to the upper (Q3) quartiles, with whiskers extending to the minimum and maximum of 1.5 times the interquartile range. Outliers are indicated by black dots. **(B)** Bar plots depict the proportion of overlapping fixations per screen half and group (healthy controls in yellow, neglect patients in gray). Standard deviations are indicated by whiskers. Significant differences are depicted by asterisks (***p < 0.001, **p < 0.01, and *p < 0.05).

As confirmed by *post hoc* tests, neglect patients produced more overlapping fixations in the right half of the screen than healthy controls (neglect patients: mean = 10.433, *SD* = 3.539; healthy controls: mean = 6.502, *SD* = 1.694; *p* < 0.001; Figure 6A). The inverse pattern was found for the left half of the screen, in which neglect patients produced fewer overlapping fixations than healthy controls (neglect patients: mean = 2.2560, *SD* = 1.828; healthy controls: mean = 6.144, *SD* = 1.770; *p* ≤ 0.001). In neglect patients, the number of overlapping fixations was significantly higher in the right compared to the left half of the screen (*p* < 0.001), whereas the number of overlapping fixations did not significantly differ between screen halves in healthy controls (*p* = 0.999).

*Post hoc* tests on the number of single fixations in the right half of the screen showed no significant difference between groups (neglect patients: mean = 3.420, *SD* = 1.391; healthy controls: mean = 4.504, *SD* = 1.213; *p* = 0.999). In the left half of the screen, fewer single fixations were found in neglect patients compared to healthy controls (neglect patients: mean = 1.594, *SD* = 1.001; healthy controls: mean = 4.357, *SD* = 1.042; *p* < 0.001). For both groups, the number of single fixations did not differ between screen halves (neglect patients, *p* = 0.072; healthy controls, *p* = 0.999).

##### Proportion of overlapping and single fixations

A mixed-model ANOVA showed significant main effects of the factors screen half (*F*_33_,_1_ = 16.214, *p* < 0.001) and group (*F*_33_,_1_ = 7.810, *p* = 0.009). The interaction between group and screen half was also significant (*F*_33_,_1_ = 14.25, *p* < 0.001), with a large effect size (η^2^ = 0.143).

Interestingly, as confirmed by *post hoc* tests, the proportion of overlapping fixations was the highest in neglect patients and within the right screen half, that is, not only significantly higher than the one within the left screen half in neglect patients (*p* < 0.001) but also significantly higher than the one in either screen halves in healthy controls (neglect patients: left = 1.495, *SD* = 0.786; right = 3.754, *SD* = 2.499; healthy controls: left = 1.568, *SD* = 0.844; right = 1.641. *SD* = 0.835; *p* < 0.010; Figure 6B).

##### Capture versus re-capture fixations

A mixed-model ANOVA showed significant main effects of the factor group (*F*_33_,_1_ = 7.223, *p* = 0.011). The interaction between group and overlapping fixation category was also significant (*F*_33_,_1_ = 15.193, *p* < 0.001), with a large effect size (η^2^ = 0.196).

**FIGURE 7:**
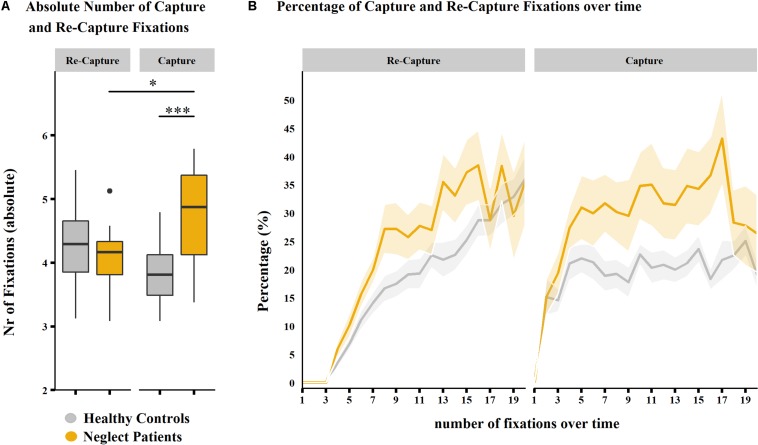
Percentage of capture and re-capture fixations over time (picture-wise analysis). **(A)** Box-and-whisker plots of the mean number of fixations per fixation category (re-capture fixations, left-hand side; capture fixations, right-hand side) in neglect patients (*n* = 15, in gray) and healthy controls (*n* = 20, in yellow). Median values per group are indicated by the horizontal black line in each box. Each box represents the lower (Q1) to the upper (Q3) quartiles, with whiskers extending from the minimum to the maximum of 1.5 times the interquartile range. Outliers are indicated by black dots. Significant differences are depicted by asterisks (^∗∗∗^*p* < 0.001 and ^∗^*p* < 0.05). **(B)** The line plot shows the mean percentage of re-capture fixations (left-hand side) and capture fixations (right-hand side) (*y*-axis) within the first 20 fixations (*x*-axis) in the group of healthy controls (in gray) and neglect patients (in yellow). The standard error of mean (SE) is depicted by semitransparent ribbons around the corresponding lines.

As confirmed by *post hoc* tests, the mean number of capture fixations was significantly higher in neglect patients than in healthy controls (mean number of capture fixations in healthy controls = 3.808, *SD* = 0.470; in neglect patients = 4.764, *SD* = 0.806; *p* < 0.001; Figure 7A). The mean number of re-capture fixations did not differ between groups (mean number of re-capture fixations in healthy controls = 4.2750, *SD* = 0.593; in neglect patients = 4.072, *SD* = 0.508; *p* = 0.999).

A qualitative illustration of the temporal dynamics shows a steep increase in the very early phase of both groups, which is more accentuated for neglect patients, reaching a plateau at approximately 30–35%. In healthy controls, the percentage of capture fixations stabilized already at a level of 20–25% (Figure 7B, right).

No significant difference between groups was found for the absolute number of re-capture fixations. The qualitative illustration of re-capture fixations showed similar temporal dynamics between groups, that is, a steady increase of re-capture fixations in the early phase of visual exploration in both groups, with a slightly steeper increase in the early phase of visual exploration in neglect patients (Figure 7B, left).

## Discussion

In the present study, we introduce a new method to analyze data collected using video-oculography during an FVE paradigm, reflecting the allocation of visual attention in space. In particular, we present the calculation of the visual exploration area, based on the physiology of foveal vision, and compare this new analysis approach with conventional analysis methods commonly used in visual attention research. We show that assessing the visual exploration area in neglect patients is a promising complementary analysis approach to evaluate visual attention in neglect patients and to accurately analyze single and overlapping fixations.

Conventional analyses, as well as our new analysis approach assessing the visual exploration area, showed visual attention allocation deficits typically observed in neglect patients (e.g., Dijkerman et al., 2003; Pflugshaupt et al., 2004; Cazzoli et al., 2009; Cazzoli et al., 2011; Delazer et al., 2018; Ohmatsu et al., 2019; Paladini et al., 2019). In comparison to healthy subjects, neglect patients showed a significant rightward horizontal shift in their mean gaze position and a significant rightward shift in the horizontal coordinates of their mean leftmost fixation. Furthermore, compared to healthy controls, neglect patients produced significantly fewer fixations and spent less time exploring the left half of the screen. As demonstrated with our new analysis approach, patients showed a significantly smaller visual exploration area within the left half of the screen compared to healthy controls. However, no difference was found regarding the visual exploration area within the right screen half. Hence, although neglect patients produced a significantly higher number of fixations within the right screen half, their visual exploration area was not increased in this part of the screen. A further analysis showed that this was due to a significant increase of overlapping fixations within the right screen half in neglect patients, which was not found in the left screen half or in either screen half in healthy controls.

In a subsequent analysis, overlapping fixations were categorized into capture fixations (i.e., successive overlapping fixation, putatively reflecting problem of impaired disengagement) and re-capture fixations (i.e., temporally distant overlapping fixations, putatively reflecting spatial working memory deficits).

Capture fixations occurred significantly more often in neglect patients than in healthy controls. Furthermore, their temporal dynamics showed a steep increase in the very early phase of both groups, which is more accentuated for neglect patients reaching a plateau at approximately 30–35%. In healthy controls, the percentage of capture fixations already stabilized at a level around 20–25% during the same time period. This result might speak for a problem of disengagement in neglect patients, that is, difficulties in shifting gaze from one spatial location toward the next one (Rafal, 1994). Theoretically, disengaging might also mean inhibiting fixation on the previous location. In other words, capture fixations might be conceptualized as a form of perseverative fixation behavior. Such an interpretation could be in line with recent findings from a previous oculomotor study obtained with a different methodological approach (Paladini et al., 2019): Neglect patients produced more perseverative fixations at adjacent locations within the right screen half during FVE, instead of first exploring the whole available visual space, even when only the early phase of visual exploration was considered. Furthermore, our interpretation would also be in line with previous studies proposing perseverative fixations (associated with a deficient response inhibition) besides delayed recancelations (associated with deficits in the spatial working memory; Mannan et al., 2005; Wansard et al., 2014).

For re-capture fixations, no significant difference was found between neglect patients and healthy controls. Considering the temporal dynamics of re-capture fixations (Figure 7B), a slightly steeper increase could be observed in the initial exploration phase in neglect patients, possibly explained by impaired spatial working memory processes (e.g., Husain et al., 2001). However, in the later phase, both groups showed a similar increase, possibly reflecting the decreasing likelihood to explore new spatial locations with increasing exploration time.

Taken together, these findings suggest that visual exploration within the right screen half is not entirely preserved in neglect patients. Besides possible impaired working memory mechanism, the present findings suggest that impaired disengagement might also play a major role, leading to an impaired shifting of gaze from one spatial location toward the next one (Rafal, 1994).

This is also in line with the findings of several previous studies concerning bilateral deficits in neglect. For instance, even though deficits in selective attention were found only within the contralesional space in neglect patients, deficits in apparent motion perception (Battelli et al., 2001) and visual short-term memory (Duncan et al., 1999) were bilateral. Furthermore, slower response to left as well as right targets with increasing neglect severity (Bartolomeo and Chokron, 1999) and a bilateral shrinkage of the available visual field under increased attentional demands (Russell et al., 2004) have been reported. Also, neglect patients were reported to show inappropriate rightward saccades (Bourgeois et al., 2015) and impaired spatial remapping following exogenous attentional shifts toward the right (Saj et al., 2019). Furthermore, in a detailed study investigating the occurrence of distinct deficit components of visual neglect, subgroups of patients were identified with variable severity combinations of working memory deficits and magnetic attraction of attention and gaze (Toba et al., 2018). In this context, impaired disengagement might be a component of the magnetic attraction of gaze phenomena observed in neglect patients. In particular, it is conceivable that, once visual fixations are attracted to the ipsilesional field, patients may have difficulties to shift gaze toward the next spatial location, possibly leading to the capture fixations observed in the present study.

Future studies may consider this new analysis approach to evaluate the portion of the picture that was effectively explored during FVE as an index of neglect severity and, over the course of time, of its recovery. Furthermore, longitudinal studies might also evaluate whether the proportion of overlapping and single fixations changes over the course of neglect recovery.

In summary, the analysis of FVE-derived eye tracking data based on the physiology of foveal vision may be a promising complementary method in visuospatial attention research to accurately measure the portion of the picture that was effectively explored and to disentangle single from overlapping fixations.

## Data Availability Statement

Individual participant data collected in this study will not be distributed openly to conform to the data privacy statements signed by our participants. However, specific aspects of the anonymized datasets and codes supporting the findings presented in this paper will be shared upon personal request.

## Ethics Statement

The studies involving human participants were reviewed and approved by the Ethics Committee Nordwest and Zentralschweiz (EKNZ), Switzerland. The patients/participants provided their written informed consent to participate in this study.

## Author Contributions

BK, TNy, and DC contributed to the conception and design of the study. BK organized the database and performed the statistical analyses. SK programmed the MATLAB code. BK and DC wrote the first draft of the manuscript. SK, TNe, and RM wrote sections of the manuscript. DC, TNy, TNe, and RM critically revised the work for important intellectual content. All authors contributed to the manuscript revision, and read and approved the submitted version.

## Conflict of Interest

The authors declare that the research was conducted in the absence of any commercial or financial relationships that could be construed as a potential conflict of interest.
